# Perception of nudge interventions to mitigate medication errors risk in healthcare service delivery

**DOI:** 10.1186/s12913-023-10247-7

**Published:** 2023-11-27

**Authors:** Keng Sheng Chew, Say Keat Ooi, Noor Fareen Abdul Rahim, Shirly Siew-Ling Wong, Vanitha Kandasamy, Shin-Shin Teo

**Affiliations:** 1 Faculty of Medicine and Health Sciences, Universiti Malaysia Sarawak, Kota Samarahan, 94300 Sarawak Malaysia; 2 Graduate School of Business, Universiti Sains Malaysia, 11800 USM, Pulau Pinang, Malaysia; 3 Faculty of Economics and Business, Universiti Malaysia Sarawak, Kota Samarahan, 94300 Sarawak Malaysia; 4 Sarawak General Hospital, Jalan Hospital, Kuching, 93586 Sarawak Malaysia; 5 Sibu Hospital, KM 5 ½, Jalan Ulu Oya, Sibu, 96000 Sarawak Malaysia

**Keywords:** Nudge interventions, Patient safety, Perceived effectiveness, Perceived ease of implementation, Acceptability

## Abstract

**Background:**

Conventional cognitive interventions to reduce medication errors have been found to be less effective as behavioural change does not always follow intention change. Nudge interventions, which subtly steer one’s choices, have recently been introduced.

**Methods:**

Conducted from February to May 2023, this study aimed to determine the relationships between perceived effectiveness and perceived ease of implementation of six nudge interventions to reduce medication errors, i.e., provider champion, provider’s commitment, peer comparison, provider education, patient education and departmental feedback, and the moderating effects of seniority of job positions and clinical experience on nudge acceptability. Partial Least Square Structural Equation Modelling was used for data analysis.

**Results and discussion:**

All six nudge strategies had significant positive relationships between perceived effectiveness and acceptability. In three out of six interventions, perceived ease of implementation was shown to have positive relationships with perceived acceptability. Only seniority of job position had a significant moderating effect on perceived ease of implementation in peer comparison intervention. Interventions that personally involve senior doctors appeared to have higher predictive accuracy than those that do not, indicating that high power-distance culture influence intervention acceptability.

**Conclusion:**

For successful nudge implementations, both intrinsic properties of the interventions and the broader sociocultural context is necessary.

**Supplementary Information:**

The online version contains supplementary material available at 10.1186/s12913-023-10247-7.

## Background

Compromises in patient safety not only may undermine the reputation of a healthcare institution [1] but may more importantly, it may lead to pertinent adverse events including death and disabilities [2]. As a result, healthcare providers and administrators are continually obligated with the responsibility of maintaining the highest possible standard of patient safety, particularly given the increasing public awareness of medical errors. While myriad factors can compromise patient safety, medication errors are identified as one of the principal causes [2].

Conventionally, the reduction of the risk of medication errors had been approached using cognitive interventions that focus solely on altering behavior through the modification of motivations and intentions [3]. This strategy assumes that behavioral change follows intention change. However, a meta-analysis conducted in 2006 showed that intention modification accounts for less than one-third of the variance observed in behavioral change [4]. Furthermore, it is recognized that people frequently rely on heuristics, mental shortcuts, and rules of thumb for daily decision-making, especially in a chaotic and stressful environment [5]. It is in these cognitively taxing situations that irrational and impulsive decisions, often leading to potential harm to patients, are more likely to be made.

In response to these challenges, a relatively recent field utilizing the concepts and applications of nudge theory has emerged [6]. Thaler and Sunstein [6] defined a nudge as an intervention aims to “gently steer a choice without forbidding the alternative options”. For example, in a systematic review by Talat et al. [7] on nudge interventions aimed to optimize medication prescribing, at total of 15 articles with 20 different types of nudge interventions were identified. The most frequently employed nudge was by modifying default settings, including inserting automatic reminders and altering the software search capabilities to display generic drug options even when brand names were searched.

Despite that, a notable gap exists in the current literature on this topic. Although numerous studies on nudge interventions to reduce medication errors had been published [7, 8], the acceptability of these nudge interventions in a healthcare setting, particularly in an Asian context, has not been adequately studied.

Acceptability of a nudge intervention is of paramount importance [9], as it has been shown that the lack of acceptability by the targeted population not only may affect its effectiveness [10] but may also hamper its implementation [11, 12]. Nonetheless, acceptability alone does necessarily translate into successful implementation as it is merely one of the antecedent assessments for successful implementation [13].

While numerous factors can influence the acceptability of a nudge intervention, two pivotal and widely studied factors are its perceived effectiveness [9–11, 14] and its perceived degree of intrusiveness [9, 10, 15]. The higher its perceived effectiveness, the greater the degree of acceptability [14] and the lower its degree of perceived intrusiveness, the greater the degree of acceptability [9].

However, in this study, perceived ease of implementation, instead of perceived intrusiveness, was included as one of the variables. This is based on a small pilot test undertaken for this current study as well as the feedback obtained, that showed that some participants had misconstrued the scoring for perceived intrusiveness. These participants mistakenly thought that the lower the score, the worse the degree of intrusiveness. Hence, to preclude this potential confusion, this current study opted to include perceived ease of implementation instead of perceived intrusiveness, even though these 2 concepts are not precisely antithetical. Hence, the objectives of this study were to investigate the relationships of perceived effectiveness as well as perceived ease of implementation on the acceptability of nudge interventions to mitigate medication error in the Malaysian healthcare setting.

## Methods

This prospective, self-administered questionnaire study was conducted from February 2023 to May 2023 to address the following research objectives, i.e., to determine the relationships between (1) perceived effectiveness and (2) perceived ease of implementation with the acceptability of six types of nudge interventions to mitigate medication errors; as well as the potential moderating effects of years of clinical experience and job positions on these relationships.

### Participants

A total of 104 clinical healthcare staff from the Emergency and Trauma Department (ETD) of Sarawak General Hospital (SGH) participated in this study. Prior informed consent was obtained for data collection. Ethics approval from the Medical Research Ethics Committee (MREC) (no: NMRR ID-22-02887-9UP) in the Malaysian National Medical Research Register website (www.nmrr.gov.my) was obtained. Participants were recruited conveniently. No personal and confidential information (such as their names, personal identification number, etc.) were collected. All participants participated voluntarily without any form of compensation.

### Materials

The conceptual framework of this study was partially drawn from the work of van Gestel et al. [9]. The independent variables in this study were (1) “perceived effectiveness” and (2) “perceived ease of implementation” and the dependent variable was acceptability of these nudge (“acceptability”) interventions. The three items from van Gestel et al. [9] included in the perceived acceptability variable for our study were (1) “How much would you accept the implementation of this measure?” (labelled “A1” in the conceptual model), (2) “How much do you appreciate the implementation of this measure?” (A2) and (3) “How much do you support the implementation of this measure?” (A3). However, this study differed from the research of van Gestel et al. [9] in 2 aspects.

First, van Gestel et al. [9] explored the nudge’s acceptability within the context of individuals’ goal alignment of long-term benefits with short-term interests. Given that the current study targets nudge interventions to reduce medication errors and to improve patient safety by healthcare staff, all participants are expected to have prioritized patient safety in alignment with their professional goals. Hence, goal alignment was not incorporated into this study’s framework.

Second, in van Gestel et al [9], the perceived intrusiveness served as one of the independent variables. As previously mentioned, perceived intrusiveness in this study was replaced with perceived ease of implementation although these two terms may not necessarily connote diametrically opposite meaning. Perceived intrusiveness can be defined as the extent to which an intervention hinders goal attainment [16], while perceived ease of implementation can be defined by the knowledge and effort necessary to initiate and maintain an intervention [17]. One item was incorporated for perceived ease of implementation (i.e., “How easy do you find to implement this measure?“) (PI1), and one item was included for perceived effectiveness (i.e., “How effective do you think this measure would be?“) (PE1). All items were rated on Likert scale of 5 ranging from 1 = “least agree” to 5 = “most agree”.

Additionally, the conceptual framework of this current study also included two additional variables: seniority of job positions (“job position”), encompassing roles from the least senior position, i.e., assistant medical officers to the most senior position, i.e., specialists, as well as years of clinical experience (“experience”). These variables, postulated to moderate the relationships between the independent variables (perceived effectiveness and perceived ease of implementation) and the acceptability of the nudges, were not part of the variables studied by van Gestel et al. [9]. “Job position” was treated as an ordinal scale ranging from 1 = assistant medical officer to 5 = specialist whereas “experience” was treated as a continuous variable.

**Fig. 1:**
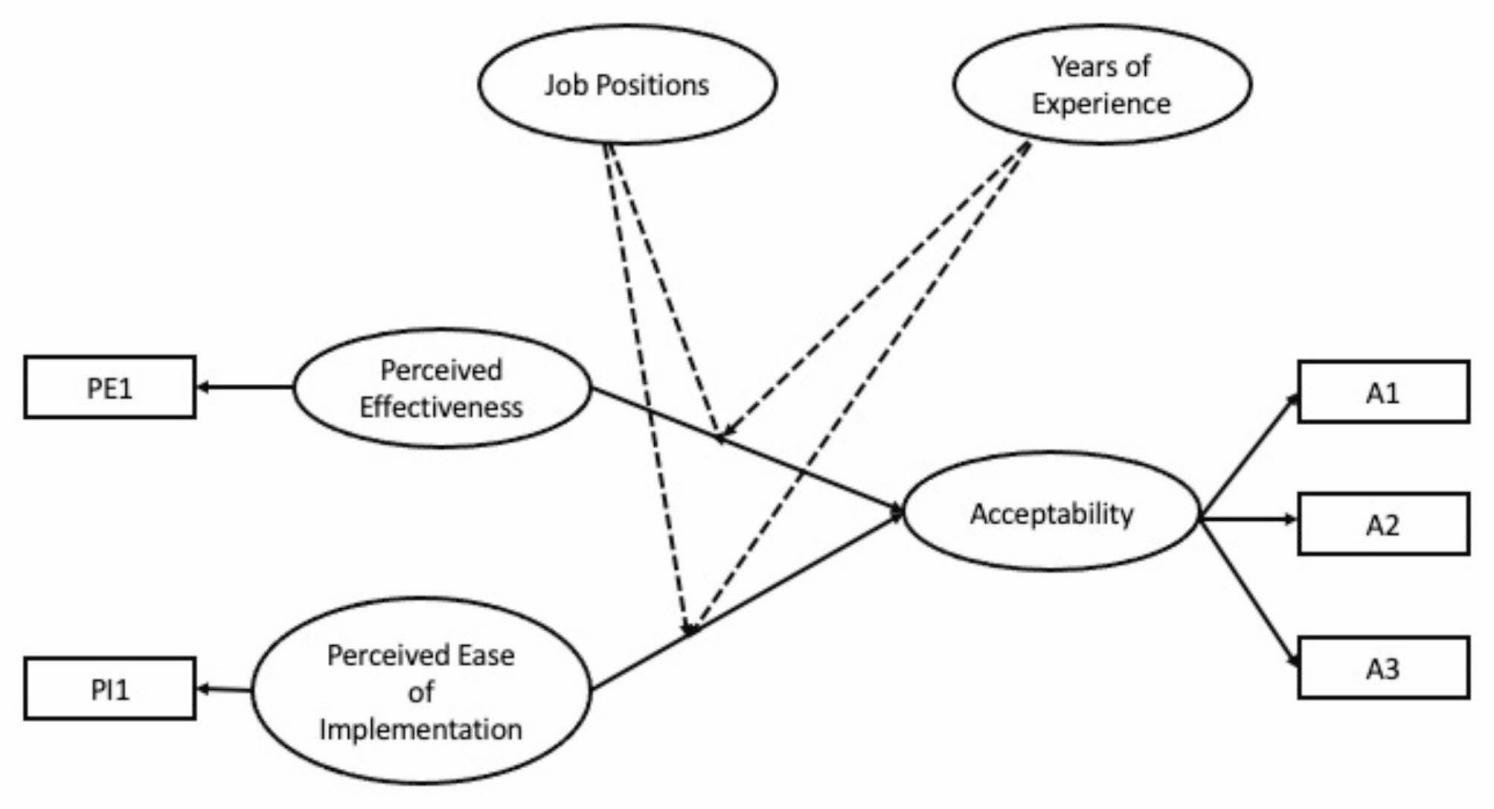
Conceptual framework of the study (the dotted lines depict moderating effect) **Note**: A1= “How much would you accept the implementation of this measure?”; A2 = “How much do you appreciate the implementation of this measure?”; A3 = “How much do you support the implementation of this measure?”; PE1 = “How effective do you think this measure would be?”; PI1= “How easy do you find to implement this measure?”

The conceptual framework of this study is given in Fig. 1. Based on this conceptual framework, the following hypotheses were tested:

1. There is a significant relationship between perceived effectiveness and the acceptability of nudge interventions (H1).
2. There is a significant relationship between perceived ease of implementation and the acceptability of nudge interventions (H2).
3. There is a significant moderating effect of seniority of job positions on the relationship between perceived effectiveness on the acceptability of nudge interventions (H3).
4. There is a significant moderating effect of seniority of job positions on the relationship between perceived ease of implementation on the acceptability of nudge interventions (H4).
5. There is a significant moderating effect of years of experience on the relationship between perceived effectiveness on the acceptability of nudge interventions (H5).
6. There is a significant moderating effect of years of experience on the relationship between perceived ease of implementation on the acceptability of nudge interventions (H6).

According to Yadav et al. [18], there are six types of nudge interventions that could be implemented to mitigate medication errors. These are (1) provider champion, (2) provider’s commitment, (3) peer comparison, (4) provider education, (5) patient education and (6) departmental feedback. The definitions of these 6 interventions are given in Table 1. In this study, only the acceptability of these 6 types of interventions [18] were analyzed, not the actual implementation. The questionnaire developed for this study is attached as Supplementary Material 1.

**Table 1:** Technical Definitions of Nudge Interventions to Mitigate Medication Errors (adapted from Yadav et al. [18])

Nudge Intervention (label)	Definitions
Provider champion (‘Champion’)	Getting experts or champions such as infectious disease physicians and nurses, microbiologists, or hospital top management to issue regular reminder messages through emails, instant messaging applications, letters, etc. and to lead good prescribing habits (such as antibiotic stewardship).
Provider’s commitment (‘Commitment’)	Doctors to sign commitment letters as pledges to practice good prescription habits. These pledges to be displayed in consultation/treatment room
Peer comparison (‘Comparison’)	Personalized performance ranking delivered through emails, letters, etc. so that each individual doctor or healthcare staff in each unit know how well they have performed compared to their peers. Doctors or teams who performed well should be given rewards such as “Top performer”, “Team of the month” awards
Departmental feedback (‘Feedback’)	Regular (e.g., monthly) departmental audit and feedback such as rate of inappropriate prescribing habits (i.e., inappropriate antibiotic use and microbial resistance patterns).
Patient Education (‘Patient Education’)	Pamphlets and posters with messages such as “Antibiotics Aren’t Always the Answers” to be displayed on the walls of the emergency department. These patient education messages are to prime patients so as not to always expect antibiotics from their doctors and to relieve the doctors from the pressure of unnecessarily prescribing antibiotics to patients.
Provider Education (‘Provider Education’)	Irrespective of their seniority, regular educational presentations highlighting important points from latest guidelines to prime doctors and nurses for good prescribing practices

### Procedures

Prior to the distribution of the questionnaire forms, a briefing on the concepts of nudge and its applications in mitigating medication errors was given by author KSC to ensure all participants have an adequate understanding of these concepts. This briefing was conducted in a hybrid mode to accommodate both physical and virtual attendees. Post-briefing, physical attendees were immediately provided with the questionnaires, all of whom consented to participate and returned the completed forms to author KSC within a half-hour time frame. For those attending the briefing via virtual means, the questionnaires (in physical forms) were subsequently disseminated by author VK, with instructions to return the completed forms within a 48-hour period, should they choose to participate. All participants eventually completed the forms and returned to the authors. Partial Least Square Structural Equation Modelling (PLS SEM) using SmartPLS version 3.0 [19] was selected for data analysis due to its ability to handle data without normality assumption and its capacity to analyze multiple regression models simultaneously. The two-stage approach of PLS-SEM proposed by Anderson and Gerbing [20] was utilized.

In the first stage, measurement modelling was conducted to evaluate the convergent validity, discriminant validity, and internal consistency reliability of the acceptance variable. Convergent validity, the degree to which multiple items measure the same variable (i.e., acceptance variable), was assessed through factor loadings and average variance extracted (AVE), with cut-off values set according to the recommendations by Hair et al. [21], i.e., factor loadings > 0.7 and AVE > 0.5. Discriminant validity was evaluated using the heterotrait-monotrait ratio of correlations (HTMT), where ideally, HTMT should be < 0.85 [22] to indicate good discriminant validity. Good internal consistency was indicated by a composite reliability of > 0.6 [23].

In the second stage, structural modelling was analyzed for hypothesis testing. Bootstrapping with 500 re-samplings was performed. The goodness of fit of the model was evaluated using the R^2^ coefficient [21]. Specifically, R^2^ is a measure of the proportion of the variance in the dependent variable that can be explained by the independent variables in the model. According to Cohen [24], R^2^ values of 0.26, 0.13, and 0.02 indicated substantial, moderate, and weak levels of predictive accuracy, respectively. The path coefficient was analyzed to determine the strength and direction of the independent variables (i.e., perceived effectiveness and perceived ease of implementation) with the dependent variable (i.e., acceptability of nudge intervention).

## Results

There were 39 (37.5%) male and 65 (62.5%) female participants in this study. The mean age of the participants was 34.2 +/- 6.2 years old and the mean years of clinical experience were 9.6 +/- 6.5 years. In terms of job positions, 10 (9.6%) were specialists, 21 (20.2%) medical officers (equivalent to residents), 14 (13.5%) were house officers (equivalent to interns), 23 (22.1%) were assistant medical officers (equivalent to medical technicians) and 36 (34.6%) were staff nurses.

The measurement modeling showed that the model had acceptable convergent validity, discriminant validity and internal consistency reliability for all six interventions. The structural modelling showed that perceived effectiveness had significant positive relationship with acceptability for all six interventions as evidenced by the path coefficients with p-value of < 0.01 (H1 was supported). On the other hand, perceived ease of implementation was shown to have significant positive relationships with acceptability for provider’s commitment, peer comparison and departmental feedback only (H2 was partially supported).

Structural modelling analysis also showed that the provider education model has the highest predictive accuracy or model fit with R^2^ = 0.75; followed by provider champion (R^2^ = 0.675), provider’s commitment (R^2^ = 0.606), peer comparison (R^2^ = 0.55), patient education (R^2^ = 0.463) and departmental feedback (R^2^ = 0.429). The Stone and Geisser’s Q^2^ value for all six interventions are greater than 0 indicating that independent variables have predictive relevance on the dependent variable [25].

With regards to the moderating effects of years of clinical experience and job position, it was found that only job position has significant moderating effect on perceived ease of implementation in peer comparison intervention, with R^2^ improvement from 0.55 (without the moderating effect) to 0.59 (with the moderating effect of job position) resulting in a small effect size [24] (only H4 was partially supported). The details of the convergent validity (i.e., factor loadings and AVE) and internal consistency reliability are given in Table 2, discriminant validity (i.e., HTMT criteria) in Table 3 and the detailed structural model results in Table 4.

**Table 2:** Results of Measurement Model

	Champion	Commitment	Comparison	Feedback	Patient Education	Provider Education
Factor loadings						
A1	0.866	0.886	0.823	0.847	0.925	0.911
A2	0.908	0.947	0.936	0.926	0.917	0.927
A3	0.896	0.952	0.948	0.902	0.940	0.923
Composite Reliability	0.920	0.949	0.930	0.921	0.948	0.943
AVE	0.793	0.862	0.817	0.796	0.859	0.847

**Table 3:** Discriminant Validity (HTMT Criterion)

Intervention 1: Champion							Intervention 4: Feedback					
	1	2	3	4	5			1	2	3	4	5
1. Acceptance							1. Acceptance					
2. Effectiveness	0.858						2. Effectiveness	0.608				
3. Experience	0.073	0.172					3. Experience	0.114	0.112			
4. Ease of implementation	0.521	0.558	0.043				4. Ease of implementation	0.402	0.466	0.131		
5. Position	0.365	0.237	0.279	0.057			5. Position	0.163	0.042	0.279	0.183	
Intervention 2: Commitment							Intervention 5: Patient Education					
	1	2	3	4	5			1	2	3	4	5
1. Acceptance							1. Acceptance					
2. Effectiveness	0.755						2. Effectiveness	0.655				
3. Experience	0.190	0.207					3. Experience	0.170	0.150			
4. Ease of implementation	0.622	0.577	0.130				4. Ease of implementation	0.481	0.493	0.107		
5. Position	0.157	0.041	0.279	0.062			5. Position	0.261	0.258	0.279	0.030	
Intervention 3: Comparison							Intervention 6: Provider Education					
	1	2	3	4	5			1	2	3	4	5
1. Acceptance							1. Acceptance					
2. Effectiveness	0.721						2. Effectiveness	0.896				
3. Experience	0.176	0.080					3. Experience	0.044	0.036			
4. Ease of implementation	0.602	0.500	0.002				4. Ease of implementation	0.596	0.665	0.028		
5. Position	0.060	0.034	0.279	0.131			5. Position	0.441	0.421	0.279	0.167	

**Table 4:** Results of the Structural Model

	Intervention 1	Intervention 2	Intervention 3	Intervention 4	Intervention 5	Intervention 6
	Champion	Commitment	Comparison	Feedback	Patient Education	Provider Education
Perceived Effectiveness	0.707* (0.128)	0.524* (0.111)	0.439* (0.119)	0.429* (0.153)	0.489* (0.184)	0.813* (0.118)
Ease of implementation	0.095 (0.143)	0.312* (0.133)	0.407* (0.117)	0.291* (0.157)	0.229 (0.206)	0.026 (0.135)
Job Position x Perceived Effectiveness	-0.021 (0.128)	-0.007 (0.114)	-0.144 (0.104)	-0.013 (0.188)	0.057 (0.168)	-0.033 (0.126)
Job Position x Perceived Ease of implementation	0.030 (0.126)	0.039 (0.118)	0.218* (0.097)	0.211 (0.144)	0.024 (0.186)	0.059 (0.118)
Experience x Perceived Effectiveness	-0.039 (0.112)	-0.036 (0.137)	0.008 (0.133)	0.083 (0.154)	0.094 (0.145)	0.082 (0.099)
Experience x Perceived Ease of implementation	0.092 (0.097)	0.083 (0.147)	-0.077 (0.117)	-0.028 (0.109)	0.008 (0.139)	-0.072 (0.102)
R^2^	0.675	0.606	0.590	0.429	0.463	0.750
Q^2^	0.500	0.495	0.437	0.306	0.363	0.607
R^2^ change of significant moderating effect			0.040 (0.0976)			
Effect Size			small			

Note: *p < 0.05

Path coefficients (std. error)

## Discussion

In summary, this study demonstrated a positive relationship between perceived effectiveness and perceived acceptability across all the nudge interventions evaluated. However, the significant association between perceived ease of implementation and perceived acceptability was identified only for the interventions concerning provider’s commitment, peer comparison, and departmental feedback. Of these interventions, the provider education model demonstrated the greatest predictive accuracy, as indicated by an R^2^ value of 0.75. Additionally, job position significantly moderated the relationship between perceived ease of implementation and acceptability in the peer comparison intervention. Collectively, these results underscore the pivotal role of perceived effectiveness as a determinant in the acceptability of nudge interventions.

Bang et al. [14] similarly emphasized that an intervention deemed beneficial and effective has a higher likelihood of successful implementation. Conversely, interventions perceived to be less effective are less likely to be acceptable for implementation [10]. Even if it is forced to be implemented, this may cause cognitive dissonance [26]. Cognitive dissonance, said to occur when an individual’s behavior conflicts with his or her personal beliefs, can result in job stress and emotional exhaustion [27]. This is particularly so in service industries marked by high degrees of intangibility, heterogeneity, inseparability, and perishability [28] such as healthcare services. As cognitive dissonance can reduce compliance and work quality [29], this emphasises the necessity for effective top-down communication in cascading information and reducing miscommunication.

Nonetheless, this study only tested perceived acceptability as the dependent variable. As previously stated, acceptability is only one of the important antecedents among an array of factors that contribute to the successful implementation of any intervention including nudges [13]. Additional variables such as appropriateness, feasibility, sustainability, and fidelity to initial objectives also play critical roles. Implementing any intervention in a real-world context is inherently challenging, a complexity that is highlighted by the Consolidated Framework for Implementation Research (CFIR). This comprehensive framework in implementation science consists of up to 39 constructs organized into five major domains: the inherent attributes of the intervention, external environmental factors or the outer setting, internal organizational characteristics or the inner setting, individual attributes of those involved in the implementation, and a variety of implementation processes ranging from planning and engagement to execution and ongoing evaluation [13].Further analysis of this study findings also revealed another more nuanced insight. This study found that nudge interventions involving senior doctors (i.e., the specialists, senior medical officers) personally, such as provider education, provider champion (i.e., having a physician as an advocate), and provider commitment, yielded a higher predictive accuracy and the model’s goodness-of-fit than nudge interventions that do not need to personally involve senior doctor, such as patient education. In these interventions that do not involve active participation of physicians (e.g. patient education and departmental feedback), it seems that perceived effectiveness alone may not be sufficient to ensure successful implementation. This suggests the probable presence of factors beyond perceived effectiveness and perceived ease of implementation that may contribute to the acceptability of these interventions.

These observations may be due to the paternalistic culture within the Malaysian healthcare system [30], and even more broadly, within the Asian setting [31]. Therefore, while empowering patients to decline inappropriate antibiotic prescriptions, for example, may be perceived as an effective nudge to remind senior doctors on the importance of good prescribing habits, this intervention may not be acceptable and feasible with the entrenched hierarchical system, where patients typically follow the doctor’s advice.

This is postulated to be due to the high power distance index (PDI) present in many Asian cultures including in Malaysia. Indeed, Malaysia is often regarded as one of the countries with highest PDI [32]. PDI is one of Hofstede’s six cultural dimensions [33] and can be defined as the extent to which less powerful members of a society accept and expect unequal power distribution favoring more powerful members. Another concept related to PDI is the concept of authority gradient [34, 35], which is prevalent in various workplace settings, including healthcare setting [34]. A steep authority gradient can stifle open communication [35], engender fear of authority [36], discourage speaking up [37] all of which may further compromise patient safety.

This postulation has significant implications for healthcare managers and policy makers. A laissez-faire leadership style, as described in Lewin’s 3-style leadership model [38], may not be suitable to ensure acceptability and successful implementation of nudge interventions in cultures with steep authority gradients. As our study suggests, in such high PDI cultures, top-down instructions or active participation of top leaders or senior doctors in leading by examples would more likely improve perceived acceptability and successful implementation of nudge interventions. In fact, the centrality of top leaders or senior doctors may also explain the significant moderating effect of job positions seen in this study on the positive relationship between ease of implementation and acceptability of peer comparison intervention.

Similarly, in Meyer’s (2014) [39] 8-axes Cultural Map, eight distinct dimensions are delineated to capture the variations in cultural practices globally. Focusing specifically on the leadership dimension, Meyer (2014) posits two extreme styles: egalitarian and hierarchical. In cultures with egalitarian leanings, there is a pervasive sense of equality that permeates the work environment, manifesting in flat organizational structures. On the opposite end, hierarchical cultures uphold a more rigid, rank-based structure where deference to higher-ranking individuals is expected. Such hierarchical organizations (exist often in Asian setting) exhibit top-down decision-making models, where decisions can be expedited if higher-ranking members invest time and resources. This is exemplified in the context of this study concerning nudge interventions, where those requiring the active engagement of senior medical professionals showed superior predictive accuracy [39].

This study has a number of pertinent limitations that need to be mentioned. First, as this study is based on self-administered questionnaires, it may suffer from self-reporting bias, i.e., participants may provide answers that they believed were desirable, rather than what they truly felt or practiced. This is particularly important given that this study involves professional practices and opinions and the fact that the participants personally knew one of the authors (KSC). Second, this study was conducted in only one center with 104 clinical staff. Given the relatively small sample size, there is a possibility that this study had limited power to detect significant moderating effects (e.g. the years of clinical experience did not appear to have any significant moderating effect). Smaller sample size may also mean that the results may not be generalizable to other hospitals, or geographic locations. Third, this study was conducted in a cross-sectional manner and as such, only provides a snapshot of opinions at that particular time. It does not capture the changes of opinion over time as well as any potential seasonal effects (such as the emergence of Coronavirus 2019 pandemic) that may influence the perceptions of the participants on nudge interventions acceptability. Fourth, the educational briefing given by author KSC on the concepts of nudge and its applications in mitigating medication errors was only a single-shot education. The quality, content, and method of such briefing may result in a limited the understanding on this topic by the participants. The fact that the briefing was conducted in hybrid mode and physical attendees opted to complete the forms there and then within 30 min after the briefing might also have potentially introduced additional discrepancies. The difference in the modality (in-person vs. virtual), for example, could have potentially resulted in discrepancies in understanding the contents of the briefing which in turn, might have affected their responses. Finally, physical attendees who opted to complete the forms within 30 min might have imposed a time pressure upon themselves. This means that they might not have sufficient time to reflect deeply on each question, which could have affected the granularity of their responses.

## Conclusion

Despite these limitations, this study suggests that adopting a holistic view is necessary when designing and implementing nudge interventions in healthcare setting in Malaysia, accounting not just for the intrinsic properties of these interventions, but also taking into consideration the broader sociocultural context in which it is deployed [28]. As healthcare services grow increasingly complex, this comprehensive approach may become ever more crucial for the successful implementation of nudge interventions aimed at improving patient safety and ultimately, hospital reputation.

### Electronic supplementary material

Below is the link to the electronic supplementary material.

Supplementary Material 1

## Data Availability

The data used maybe obtained by contacting the corresponding author.
