# Supplementary material for: Perception of nudge interventions to mitigate medication errors risk in healthcare service delivery
Source: BMC Health Serv Res. 2023 Nov 27;23:1310. doi: 10.1186/s12913-023-10247-7 (PMC10683206; doi:10.1186/s12913-023-10247-7)
Supplement: Supplementary file 1 — Supplementary Material 1 [file 12913_2023_10247_MOESM1_ESM.docx]

# **Questionnaire**

#

| Gender:   - Male - Female | Age: ­­­­  ____ years | Years of clinical experience:  ____ years |
| --- | --- | --- |
| Job designation: |  |  |
| - Specialist - Medical Officer - House Officer | - Staff nurse - Assistant Medical Officer |  |
|  | | |
| **Nudge Intervention 1:**  Getting experts or champions such as infectious disease physicians and nurses, microbiologists, or hospital top management to issue regular reminder messages through emails, instant messaging applications, letters, etc. and to lead good prescribing habits (such as antibiotic stewardship). | | |
| “How much would you accept the implementation of this measure?” | | \| Least likely \|  \|  \|  \| Most likely \| \| --- \| --- \| --- \| --- \| --- \| \| 1 \| 2 \| 3 \| 4 \| 5 \| |
| “How much do you appreciate the implementation of this measure?” | | \| Least likely \|  \|  \|  \| Most likely \| \| --- \| --- \| --- \| --- \| --- \| \| 1 \| 2 \| 3 \| 4 \| 5 \| |
| “How much do you support the implementation of this measure?” | | \| Least likely \|  \|  \|  \| Most likely \| \| --- \| --- \| --- \| --- \| --- \| \| 1 \| 2 \| 3 \| 4 \| 5 \| |
| “How effective do you think this measure would be?” | | \| Least likely \|  \|  \|  \| Most likely \| \| --- \| --- \| --- \| --- \| --- \| \| 1 \| 2 \| 3 \| 4 \| 5 \| |
| “How easy do you find to implement this measure”? | | \| Least likely \|  \|  \|  \| Most likely \| \| --- \| --- \| --- \| --- \| --- \| \| 1 \| 2 \| 3 \| 4 \| 5 \| |
|  | |  |
| **Nudge Intervention 2:**  Doctors to sign commitment letters as pledges to practice good prescription habits. These pledges to be displayed in consultation/treatment room. | | |
| “How much would you accept the implementation of this measure?” | | \| Least likely \|  \|  \|  \| Most likely \| \| --- \| --- \| --- \| --- \| --- \| \| 1 \| 2 \| 3 \| 4 \| 5 \| |
| “How much do you appreciate the implementation of this measure?” | | \| Least likely \|  \|  \|  \| Most likely \| \| --- \| --- \| --- \| --- \| --- \| \| 1 \| 2 \| 3 \| 4 \| 5 \| |
| “How much do you support the implementation of this measure?” | | \| Least likely \|  \|  \|  \| Most likely \| \| --- \| --- \| --- \| --- \| --- \| \| 1 \| 2 \| 3 \| 4 \| 5 \| |
| “How effective do you think this measure would be?” | | \| Least likely \|  \|  \|  \| Most likely \| \| --- \| --- \| --- \| --- \| --- \| \| 1 \| 2 \| 3 \| 4 \| 5 \| |
| “How easy do you find to implement this measure”? | | \| Least likely \|  \|  \|  \| Most likely \| \| --- \| --- \| --- \| --- \| --- \| \| 1 \| 2 \| 3 \| 4 \| 5 \| |
|  | | |
| **Nudge Intervention 3:**  Personalized performance ranking delivered through emails, letters, etc. so that each individual doctor or healthcare staff in each unit know how well they have performed compared to their peers. Doctors or teams who performed well should be given rewards such as “Top performer”, “Team of the month” awards. | | |
| “How much would you accept the implementation of this measure?” | | \| Least likely \|  \|  \|  \| Most likely \| \| --- \| --- \| --- \| --- \| --- \| \| 1 \| 2 \| 3 \| 4 \| 5 \| |
| “How much do you appreciate the implementation of this measure?” | | \| Least likely \|  \|  \|  \| Most likely \| \| --- \| --- \| --- \| --- \| --- \| \| 1 \| 2 \| 3 \| 4 \| 5 \| |
| “How much do you support the implementation of this measure?” | | \| Least likely \|  \|  \|  \| Most likely \| \| --- \| --- \| --- \| --- \| --- \| \| 1 \| 2 \| 3 \| 4 \| 5 \| |
| “How effective do you think this measure would be?” | | \| Least likely \|  \|  \|  \| Most likely \| \| --- \| --- \| --- \| --- \| --- \| \| 1 \| 2 \| 3 \| 4 \| 5 \| |
| “How easy do you find to implement this measure”? | | \| Least likely \|  \|  \|  \| Most likely \| \| --- \| --- \| --- \| --- \| --- \| \| 1 \| 2 \| 3 \| 4 \| 5 \| |
|  | | |
| **Nudge Intervention 4:**  Regular (e.g., monthly) departmental audit and feedback such as rate of inappropriate prescribing habits (i.e., inappropriate antibiotic use and microbial resistance patterns). | | |
| “How much would you accept the implementation of this measure?” | | \| Least likely \|  \|  \|  \| Most likely \| \| --- \| --- \| --- \| --- \| --- \| \| 1 \| 2 \| 3 \| 4 \| 5 \| |
| “How much do you appreciate the implementation of this measure?” | | \| Least likely \|  \|  \|  \| Most likely \| \| --- \| --- \| --- \| --- \| --- \| \| 1 \| 2 \| 3 \| 4 \| 5 \| |
| “How much do you support the implementation of this measure?” | | \| Least likely \|  \|  \|  \| Most likely \| \| --- \| --- \| --- \| --- \| --- \| \| 1 \| 2 \| 3 \| 4 \| 5 \| |
| “How effective do you think this measure would be?” | | \| Least likely \|  \|  \|  \| Most likely \| \| --- \| --- \| --- \| --- \| --- \| \| 1 \| 2 \| 3 \| 4 \| 5 \| |
| “How easy do you find to implement this measure”? | | \| Least likely \|  \|  \|  \| Most likely \| \| --- \| --- \| --- \| --- \| --- \| \| 1 \| 2 \| 3 \| 4 \| 5 \| |
|  | | |
| **Nudge Intervention 5:**  Pamphlets and posters with messages such as “Antibiotics Aren’t Always the Answers” to be displayed on the walls of the emergency department. These patient education messages are to prime patients so as not to always expect antibiotics from their doctors and to relieve the doctors from the pressure of unnecessarily prescribing antibiotics to patients. | | |
| “How much would you accept the implementation of this measure?” | | \| Least likely \|  \|  \|  \| Most likely \| \| --- \| --- \| --- \| --- \| --- \| \| 1 \| 2 \| 3 \| 4 \| 5 \| |
| “How much do you appreciate the implementation of this measure?” | | \| Least likely \|  \|  \|  \| Most likely \| \| --- \| --- \| --- \| --- \| --- \| \| 1 \| 2 \| 3 \| 4 \| 5 \| |
| “How much do you support the implementation of this measure?” | | \| Least likely \|  \|  \|  \| Most likely \| \| --- \| --- \| --- \| --- \| --- \| \| 1 \| 2 \| 3 \| 4 \| 5 \| |
| “How effective do you think this measure would be?” | | \| Least likely \|  \|  \|  \| Most likely \| \| --- \| --- \| --- \| --- \| --- \| \| 1 \| 2 \| 3 \| 4 \| 5 \| |
| “How easy do you find to implement this measure”? | | \| Least likely \|  \|  \|  \| Most likely \| \| --- \| --- \| --- \| --- \| --- \| \| 1 \| 2 \| 3 \| 4 \| 5 \| |
|  | | |
| **Nudge Intervention 6:**  Irrespective of their seniority, regular educational presentations highlighting important points from latest guidelines to prime doctors and nurses for good prescribing practices | | |
| “How much would you accept the implementation of this measure?” | | \| Least likely \|  \|  \|  \| Most likely \| \| --- \| --- \| --- \| --- \| --- \| \| 1 \| 2 \| 3 \| 4 \| 5 \| |
| “How much do you appreciate the implementation of this measure?” | | \| Least likely \|  \|  \|  \| Most likely \| \| --- \| --- \| --- \| --- \| --- \| \| 1 \| 2 \| 3 \| 4 \| 5 \| |
| “How much do you support the implementation of this measure?” | | \| Least likely \|  \|  \|  \| Most likely \| \| --- \| --- \| --- \| --- \| --- \| \| 1 \| 2 \| 3 \| 4 \| 5 \| |
| “How effective do you think this measure would be?” | | \| Least likely \|  \|  \|  \| Most likely \| \| --- \| --- \| --- \| --- \| --- \| \| 1 \| 2 \| 3 \| 4 \| 5 \| |
| “How easy do you find to implement this measure”? | | \| Least likely \|  \|  \|  \| Most likely \| \| --- \| --- \| --- \| --- \| --- \| \| 1 \| 2 \| 3 \| 4 \| 5 \| |
|  | | |
